# The myxocoumarins A and B from *Stigmatella aurantiaca* strain MYX-030

**DOI:** 10.3762/bjoc.9.293

**Published:** 2013-11-20

**Authors:** Tobias A M Gulder, Snežana Neff, Traugott Schüz, Tammo Winkler, René Gees, Bettina Böhlendorf

**Affiliations:** 1 Kekulé Institute of Organic Chemistry and Biochemistry, University of Bonn, Gerhard-Domagk-Straβe 1, 53121 Bonn, Germany; 2 Syngenta Crop Protection AG, CH-4002 Basel, Switzerland

**Keywords:** antifungal activity, myxobacteria, natural products, *Stigmatella aurantiaca*, structure elucidation

## Abstract

The myxobacterial strain *Stigmatella aurantiaca* MYX-030 was selected as promising source for the discovery of new biologically active natural products by our screening methodology. The isolation, structure elucidation and initial biological evaluation of the myxocoumarins derived from this strain are described in this work. These compounds comprise an unusual structural framework and exhibit remarkable antifungal properties.

## Introduction

**Figure 1:**
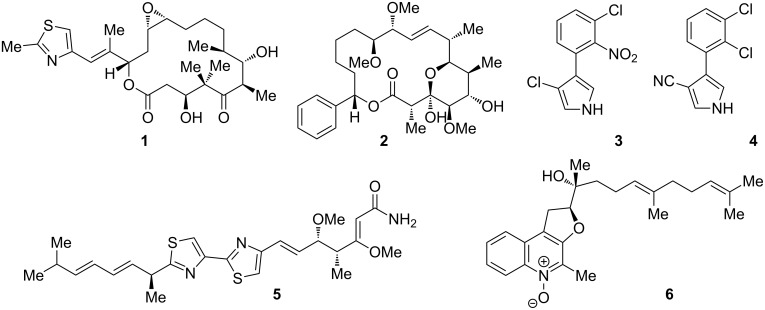
Structures of epothilone A (**1**), soraphen A_1α_ (**2**), pyrrolnitrin (**3**), fenpiclonil (**4**), myxothiazol A (**5**), and aurachin A (**6**).

Despite declining interest of most big R&D-driven chemical companies in recent years, natural products continue to serve as one of the most important sources of new bioactive chemical entities, both in the pharmaceutical [1–4] as well as in the agrochemical industry [5–8]. A particularly rich source of intriguing secondary metabolites with interesting biological properties are the myxobacteria [9–12]. These organisms are especially talented in assembling PKS-, NRPS- and PKS/NRPS-hybrid products, often incorporating unusual biochemistry in the respective biosynthetic pathways [13–15]. The most well-known myxobacterial natural products are the epothilones (e.g. epothilones A (**1**), Figure 1), microtubule-stabilizing macrolactones that are clinically used in cancer therapy [16–20].

But also from an agrochemical point of view, myxobacteria have already furnished a large set of promising lead structures, in particular in the context of antifungal compounds with around 50% of myxobacteria-derived natural products exhibiting such properties [10]. This activity of myxobacterial metabolites results from diverse modes of action, such as inhibition of DNA replication, transcription and translation, as well as interference with cell membrane stability [21]. Inhibition of acetyl-CoA carboxylase is exerted by soraphen A_1α_ (**2**), which has been shown to be a potent and broad spectrum fungicide [22–23]. The most dominant target of myxobacterial antifungal agents, however, is the mitochondrial respiratory chain [21]. The compounds thereby either target complex I (NADH dehydrogenase) of the electron transport chain, as for example pyrrolnitrin (**3**) [24–25], which is in use for the treatment of fungal skin infections [26] and served as a lead structure for the development of the agrochemical agents fenpiclonil (**4**) and fludioxonil [27], or complex III (cytochrome bc_1_), as for example myxothiazol A (**5**) [28–29]. Compounds of the same structural family can also target different target complexes simultaneously, as for example in the case of aurachins (e.g. aurachin A (**6**), Figure 1) [30–32]. Due to the remarkably high percentage of antifungal myxobacterial natural products, we investigated the potential of new myxobacterial isolates towards the production of novel, agrochemically relevant antifungal lead structures. The results obtained with strain *Stigmatella aurantiaca* MYX-030 are described herein.

## Results and Discussion

The myxobacterial strain *Stigmatella aurantiaca* MYX-030 has been selected as interesting producer of bioactive natural products by our standardized hit follow-up process. Initial dereplication of known compounds was performed by using HPLC–UV–MS [33] combined with Peak–Activity–Correlation (PAC) data. Taken together these results indicated the presence of the known antifungal myxobacterial compound myxothiazole A (**5**), as well as the aurachins A (**6**) and C in the extract. In addition to these metabolites, a series of non-polar compounds exhibiting strong antifungal activity was detected. Comparison of UV and molecular mass data of these secondary metabolites with an in-house compound library resulted in no hits, thus prompting us to their isolation and structural elucidation.

The UV spectrum of **7** revealed maxima at 218 and 286 nm, strongly suggesting the presence of an aromatic system within the structure. Negative mode ESIMS analysis of the most abundant congener **7** of this set of natural products showed strong signals at 378.2 ([M − H]^−^) and 757.4 ([2M − H]^−^) as well as a positive ESIMS signal at 362.2 ([M − H_2_O + H]^+^). High resolution positive mode ESI–TOF mass analysis resulted in a molecular mass of 380.2097 u, which best fits a calculated molecular formula of C_20_H_30_NO_6_ for the [M + H]^+^ ion of **7**, thus indicating 7 degrees of unsaturation. The calculated number of carbon atoms was further substantiated by the 20 resolved signals observed in the compound’s ^13^C NMR spectrum (Table 1). These signals were assigned to three methyl groups (14.0, 16.5, and 21.2 ppm), eight methylene units (22.6, 23.4, 29.2, 29.3, 29.4, 29.8, 31.8, and 38.3 ppm), a quaternary carbon at 46.5 ppm, a heteroatom-substituted quaternary carbon – most likely a tertiary alcohol – at 79.1 ppm, an ester-type functionality at 172.5 ppm, and six sp^2^-hybridized carbon atoms as part of an highly heteroatom-substituted aromatic system (103.2, 108.0, 118.6, 148.3, 149.2, and 157.8 ppm). The presence of the latter moiety was further corroborated by the ^1^H NMR spectral data: two doublets with chemical shifts at 7.43 and 7.35 ppm and a coupling constant of 2 Hz clearly evidenced a highly substituted aromatic ring system bearing two protons in meta- position to each other.

**Table 1:** NMR data of compound **7** at 500 (^1^H) and 150 (^13^C) MHz.

Position	δ_C_, type^a^	δ_H_ (*J* in Hz)^b^	HMBC^b^

1			
2	172.5, C		
3	46.5, C		
4	79.1, C		
4’	118.6, C		
5	157.8, C		
6	108.0, CH	7.43, d (2)	4’, 5, 7, 8
7	148.3, C		
8	103.2, CH	7.35, d (2)	4’, 7, 8’
8’	149.2, C		
9	38.3, CH_2_	1.56, td 1.99, td	3, 4, 4’, 10, 11 3, 4, 4’, 10, 11
10	23.4, CH_2_	1.49, m 1.08, m	
11	29.2*, CH_2_	1.14-1.30, m	
12	29.3*, CH_2_	1.14-1.30, m	
13	29.4*, CH_2_	1.14-1.30, m	
14	29.8*, CH_2_	1.14-1.30, m	
15	31.8, CH_2_	1.14-1.30, m	
16	22.6, CH_2_	1.14-1.30, m	
17	14.0, CH_3_	0.86, t (7)	15, 16
18	16.5, CH_3_	1.33, s	2, 3, 4, 19
19	21.2, CH_3_	1.18, s	2, 3, 4, 18

^a^Recorded in CDCl_3_ containing a drop of CD_3_OD; ^b1^H, HSQC and HMBC data recorded in CD_3_OD. *Signal assignment interchangeable.

**Figure 2:**
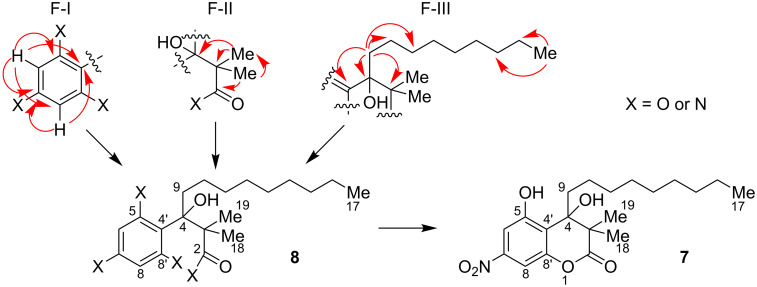
HMBC interactions used for the structure elucidation of myxocoumarin A (**7**).

The proton signals at 7.43 and 7.35 ppm correlated in an HSQC experiment with the ^13^C NMR signals at 108.0 and 103.2 ppm, respectively. The structural organization of the aromatic system was further investigated by HMBC spectroscopy. The proton at 7.43 ppm showed strong correlations with two of the heteroatom-substituted carbons at 157.8 and 148.3 ppm, the putatively alkyl substituted position at 118.6 ppm, as well as the C atom at 103.2 ppm. The proton at 7.35 ppm, in turn, correlated with the C atoms at 149.2, 148.3, and 118.6 ppm, leading to overall structural fragment I (F-I) shown in Figure 2. In addition to the aromatic protons, three methyl signals were observed in the ^1^H NMR spectra: a triplet at 0.86 ppm (^3^*J* = 7 Hz), suggesting a methyl group directly attached to a CH_2_-unit, as well as two singlets at 1.18 and 1.33 ppm. Furthermore, a pair of diastereomeric protons at 1.56 and 1.99 (higher order multiplets with a triplet of doublets structure) and a set of diastereomeric protons at 1.08 and 1.49 ppm (m) were observed. The signals of the other six methylene units formed a multiplet at 1.14–1.30 ppm, indicating the presence of a linear alkyl chain in the molecule. This is corroborated by the HSQC correlations to the C atoms at 29 ppm, as well as 22.6 and 31.8 ppm. In the HMBC data, strong correlations of the isolated methyl groups to each other, to the putative ester functionality, the quaternary carbon at 46.5 ppm, and the tertiary alcohol carbon at 79.1 ppm led to the assembly of structural fragment II (F-II, Figure 2). Further substitution of the latter was obvious from HMBC cross peaks of the diastereotopic CH_2_ group (1.56 and 1.99 ppm) to the latter position (79.1 ppm) and to the carbon bearing the two methyl groups (46.5 ppm), as well as to the aromatic carbon at 118.6 ppm. In addition, this methylene unit exhibited correlations with two further CH_2_ groups of the linear alkyl chain. This side chain turned out to consist of 8 neighboring CH_2_ units with the methyl functionality at 0.86 ppm as the end group, as evident from HMBC correlations of the latter to the final two methylene units of this chain (F-III, Figure 2). Taken together, this information allowed the assembly of all aforementioned structural fragments to give a draft structure **8**. Given the chemical shifts of the carbonyl C-2 (172.5 ppm) and of carbon C-8’ (149.2 ppm), the presence of a lactone moiety can be deduced. The resulting overall structure still lacks three oxygens, a hydrogen, and a nitrogen atom combined with one degree of unsaturation when compared to the calculated molecular formula of **7**. Consequently, the substituents at C-5 and C-7 can be identified as an OH and a nitro function, respectively, as also indicated by the chemical shifts of C-5 (157.8 ppm) and C-7 (148.3 ppm). This results in the final overall structure **7** of this natural product. To unambiguously prove the substitution pattern at the aromatic ring system, the phenolic OH-group was selectively methylated using diazomethane. The resulting methyl ether was irradiated in a 1D NOE experiment, which resulted in the expected strong increase of the proton bound to C-6. This observation thus firmly validated that the methyl ether had to be situated at C-5, as its alternative location at C-7 would have led to a strong increase in signal intensity of the protons bound to both, C-6 and C-7.

**Figure 3:**
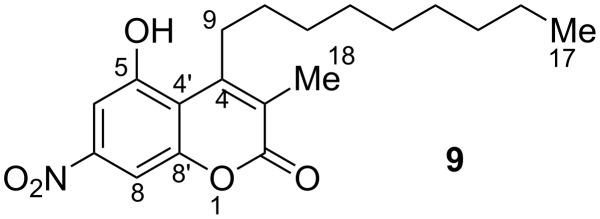
Chemical structure of myxocoumarin B (**9**).

Owing to its coumarin core structure and its biological origin, compound **7** was named myxocoumarin A (**7**). Besides **7**, a second metabolite **9** with a highly similar NMR spectrum was isolated. ESIMS analysis of **9** showed a molecular ion peak at 346.2 ([M − H]^−^, negative mode) and 348.2 ([M + H]^+^, positive mode), consistent with a formal loss of MeOH when compared to the molecular mass of myxocoumarin A (**7**). In addition, no loss of water was observable in the MS data, suggesting the hydroxy function at C-4 in **7** not to be present in **9**. Comparison of the ^1^H NMR spectra of **7** and **9** furthermore revealed the missing signals of the methyl groups 18 and 19 of **7** in the spectrum of **9**, along with a new singlet at 2.20 ppm integrating for three protons. In conclusion, this lead to the proposed structure of myxocoumarin B (**9**) as shown in Figure 3, which was unambiguously verified by in-depth analysis of chemical shifts in comparison with literature data (see Supporting Information File 1).

Unfortunately, the amount of **7** isolated from the initial fermentation broth was not sufficient for stereochemical investigations, nor did the obtained amount of **9** suffice to obtain high quality ^13^C NMR data. Attempts to produce more crude material were not successful, as cryo cultures of the producing strain failed to be transformed into actively growing cultures again.

Coumarins in general constitute a large group of bioactive natural products, mostly isolated from plants. Most of these plant-derived compounds are polyphenols that are often further modified by most diverse functionalization reactions, such as *O*-methylation, glycosylation, prenylation, oxygenation and subsequent cyclization or dimerization events [34]. Nitrogen-bearing congeners are rather scarce, with the bacterial aminocoumarins, such as the gyrase inhibitor novobiocin, being the most well-known examples [35]. Despite the larger number of existing coumarin structures in literature, the 5-hydroxy-7-nitro substitution pattern combined with the unusual long-chain, fully saturated alkyl substituent at C-4 are unique to the myxcoumarins **7** and **9**.

**Scheme 1:**
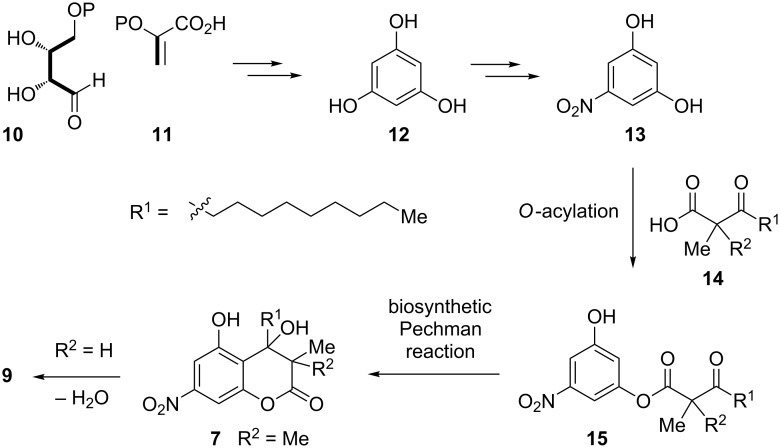
Postulated biosynthetic pathway to myxocoumarins A (**7**) and B (**9**).

With the production of myxothiazol A (**5**) and aurachin A (**6**), the investigated strain *Stigmatella aurantiaca* MYX-030 resembles the secondary metabolite profile of previously chemically investigted *S. aurantiaca* and *S. erecta* strains. Interestingly, both, *S. aurantiaca* Sg a15 and *S. erecta* Pd e21, were reported to produce 5-nitroresorcinol (**13**) [36]. The biosynthesis of **13** was investigated in *S. erecta* Pd e21 using isotope-labeling experiments that revealed its precursor molecules to be glucose-derived erythrose-4-phosphate (**10**) and phosphoenol-pyruvate (**11**, Scheme 1). Due to the observed scrambling of the expected labeling pattern in **13**, its biosynthesis has to proceed via a symmetrical intermediate, putatively phloroglucinol (**12**) [36]. Nitrophenol **13** likely constitutes a direct biosynthetic precursor of the myxocoumarins. Upon *O*-acetylation of **13** with the long-chain β-keto acid building block **14**, putatively recruited from fatty-acid biosynthesis, the intermediate ester **15** could undergo C–C bond formation by nucleophilic attack of the aromatic system to the side-chain keto functionality, comparable to the first step of a Pechman condensation reaction. This would already furnish mycoxoumarin A (**7**, R^2^ = Me), with myxocoumarin B (**9**, for R^2^ = H) being formed after additional loss of H_2_O. In order to identify further myxocoumarin producing strains, chemical screenings of the secondary metabolite profiles of *S. aurantiaca* Sg a15 and *S. erecta* Pd e21 using the fermentation conditions identified for *S. aurantiaca* MYX-030 might thus be worthwhile.

To verify the initially measured antifungal activity from the PAC, myxocoumarin A (**7**) was subjected to leaf disk assays against a series of agriculturally relevant pathogenic fungi. The compound revealed interesting antifungal activities with complete inhibition of agronomically important pathogens such as *Botrytis cinerea* at 200 mg/L as well as *Magnaporthe grisea* and *Phaeosphaeria nodorum* at 67 mg/L. Partial effects were found against *Blumeria graminis* (67 mg/L). The compound was, however, inactive against *Drechslera teres* and *Phytophthora infestans*. These findings indicate a broad range antifungal activity of **7**, comparable to commercial standard fungicides, although on a somewhat lower activity level. To further substantiate these results, liquid culture assays against a set of fungal pathogens were performed, showing strong intrinsic antifungal activities against *M. grisea* (100% inhibition at 0.7 mg/L) as well as *B. cinerea*, *Fusarium culmorum*, and *P. nodorum* (all at 2 mg/L), with no activity against *Pythium ultimum* and *Rhizoctonia solani*. So far, no insecticidal and herbicidal activities have been observed. Due to the problems arising from the lacking culturability of the producing strain *S. aurantiaca*, further tests with **7** were so far not possible. Structure–activity relationship (SAR) studies on synthetic myxocoumarin derivatives as well as in-depth investigation of their antibacterial potential are currently performed in our laboratory and will be reported in due course.

## Experimental

**Cultivation and extraction.** The preculture of *S. aurantiaca* MYX-030, inoculated directly from a single culture of an agar plate, was incubated by shaking (150 rpm) at 30 °C for 7 days in a 500 mL shake flask without baffle containing 80 mL of medium MIX-5 (composition: 2 g glucose, 5 g potato starch, 2 g peptone, 0.5 g MgSO_4_, 0.5 g CaCl_2_, 10 g HEPES for 1 L H_2_O, pH 7.4). For production of myxocoumarins, 3 × 200 mL production cultures in 500 mL flasks without baffle were inoculated with 20 mL of the preculture. Production was carried out by shaking (150 rpm) at 30 °C for 10 days in MIX-1 media (composition: 5 g casitone, 2 g starch, 2 g MgSO_4_, 0.5 g CaCl_2_, 1 mL mineral solution (consisting of 0.1 g H_3_BO_3_, 5 g FeSO_4_·7H_2_O, 0.05 g KI, 2 g CoCl_2_·6H_2_O, 0.2 g CuSO_4_·5H_2_O, 2 g MnCl_2_·4H_2_O, 4 g ZnSO_4_·7H_2_O, 1 g 95% H_2_SO_4_ for 1 L solution), 10 mL vitamin solution (consisting of 10 mg folic acid, 6 mg biotin, 0.2 g *p*-aminobenzoic acid, 1 g thiamin·HCl, 1.2 g panthothenic acid, 1 g riboflavin, 2.3 g nicotinic acid, 1.2 g pyridoxine·HCl, 0.1 g vitamin B12 for 1 L solution) for 1 L H_2_O, pH 7.4) supplemented with 10% XAD-16 for continued product absorption during fermentation. Cells and XAD-16 were separated from the culture broth by filtration and subsequently extracted using methanol and acetone.

**Isolation of mycoxoumarins A (7) and B (9).** The crude material obtained by extraction of bacterial cells and XAD (341 mg) was subjected to chromatography on Sephadex LH-20 (3 × 69 cm column, flow: 1.6 mL/min) using methanol as the eluent. The myxocoumarin containing fraction (12.1 mg) was purified by reversed-phase preparative HPLC (Bischoff Kromasil 100 C-18, 10 µm, 2 cm diameter, 20 cm length) using H_2_O + 0.1% TFA (solvent A) and acetonitrile (ACN) + 0.1% TFA (solvent B) as the eluents at a flow rate of 10 mL/min using the following linear gradient: 0 min 80% A, 2 min 80% A, 20 min 50% A, 25 min 20% A. Myxocoumarin A (**7**) was collected at a retention time of 12.5 min and myxocoumarin B (**9**) at 14.7 min to give 1.9 mg and 0.5 mg pure material, respectively.

Myxocoumarin A (**7**): pale yellow solid, [α]_D_^ 20^ −6.3 (*c* 0.2, methanol); ^1^H and ^13^C NMR data, see Table 1; UV (H_2_O (64%)/ACN (36%) + 0.1% TFA, online) λ_max_, nm: 218, 286, 340 (sh); ESIMS(−) *m*/*z*: 57.4 [2M − H]^−^, 378.2 [M − H]^−^; ESIMS(+) *m*/*z* 362.2 [M − H_2_O + H]^+^; HRMS–ESI(+) *m*/*z*: [M + H]^+^ calcd. for C_20_H_30_NO_6_, 380.207; found, 380.2097 (calculated for).

Myxocoumarin B (**9**): pale yellow solid, ^1^H NMR (500 MHz, CD_3_OD) δ_H_ 7.55 (d, *J* = 2 Hz, 1H, H-6), 7.51 (d, *J* = 2 Hz, 1H, H-8), 3.18 (m, 2H, H-9), 2.20 (s, 3H, H-18), 1.62 (m, 2H, H-10), 1.49 (m, 2H, H-11), 1.42–1.26 (m, 10H, H-12, H-13, H-14, H-15, H-16), 0.90 (t, *J* = 7 Hz, 3H, H-17); UV (H_2_O (61%)/ACN (39%) + 0.1% TFA, online) λ_max_, nm: 233, 260, 332 (sh); ESIMS(−) *m*/*z*: 693.3 [2M − H]^−^, 346.2 [M − H]^−^; ESIMS(+) *m*/*z*: 348.2 [M + H]^+^.

**Biological testing.** Cut leaf disks (15 mm^2^) of the respective host plants (see below) were placed on water agar in multi-well plates (24-well format) and sprayed with 12 µL aqueous test solution of varying concentrations (dose range 22–200 mg/L) using automated microspray technology. After drying, the leaf disks were inoculated with a spore suspension of the respective pathogenic fungus (see below). The activity of myxocoumarin A (**7**) was assessed 4–6 days after inoculation as percent preventive antifungal activity as compared to the non-treated controls. The following host/pathogen combinations were used: *Phytophthora infestans* (Mont) de Bary (late blight) on tomato; *Botrytis cinerea* Pers.: Fr. (gray mould) on bean; *Blumeria graminis* f.sp. *hordei* (DC.) Speer (powdery mildew) on barley; *Drechslera teres* (Sacc.) Shoemaker (net blotch) on barley; *Phaeosphaeria nodorum* (E. Müll.) Hedjar. (glume blotch) on wheat; *Magnaporthe grisea* (T.T. Hebert) M.E. Barr(rice blast) on rice.

The intrinsic antifungal activity of the compounds was tested in liquid culture assays. Mycelial fragments prepared from a fresh liquid culture (*Pythium ultimum* Trow; *Rhizoctonia solani* Kühn) or conidia of the fungus from cryogenic storage (*B. cinerea*; *P. nodorum*; *P. grisea*; *Fusarium culmorum* (Wm. G. Sm.) Sacc.) were directly mixed into PDB potato dextrose broth. 10 µL test solution in DMSO (2%) of different concentration of **7** was placed into microtiter plates (96-well format) and the nutrient broth (90 µL) containing the fungal cells was added. The test plates were incubated at 24 °C and percent inhibition of fungal growth was determined photometrically after 48 h (*P. ultimum*; *F. culmorum*; *R. solani*) or 72 h (*B. cinerea*; *P. nodorum*; *P. grisea*) in relation to the non-treated control.

## Supporting Information

File 1 NMR and MS spectra of myxocoumarin A (**7**) and B (**9**). In-depth discussion and analysis of chemical shifts for the verification of the structure of **9**.
