# Supplementary material for: The myxocoumarins A and B from Stigmatella aurantiaca strain MYX-030
Source: Beilstein J Org Chem. 2013 Nov 20;9:2579–85. doi: 10.3762/bjoc.9.293 (PMC3869339; doi:10.3762/bjoc.9.293)
Supplement: File 1 — NMR and MS spectra of myxocoumarin A (7) and B (9). In-depth discussion and analysis of chemical shifts for the verification of the structure of 9. [file Beilstein_J_Org_Chem-09-2579-s001.pdf]

Supporting Information

for

The myxocoumarins A and B from *Stigmatella aurantiaca* strain MYX-030

Tobias A. M. Gulder*[1], Snežana Neff[2], Traugott Schüz[2], Tammo Winkler[2], René Gees[2] and Bettina Böhlendorf*[2]

Address: [1]Kekulé Institute of Organic Chemistry and Biochemistry, University of Bonn, Gerhard-Domagk-Straβe 1, 53121 Bonn, Germany and [2]Syngenta Crop Protection AG, CH-4002 Basel, Switzerland

Email: Tobias A. M. Gulder* - tgulder@uni-bonn.de; Bettina Böhlendorf* - bettina.boehlendorf@dsm.com

*Corresponding author

This work is dedicated to Prof. Dr. Gerhard Höfle on the occasion of his 74th birthday.

NMR and MS spectra of myxocoumarin A (7) and B (9). In-depth discussion and analysis of chemical shifts for the verification of the structure of 9

Table of contents:

^{1}H NMR spectrum of myxocoumarin A (**7**)	Page S3
^{13}C NMR spectrum of **7**	Page S4
HSQC spectrum of **7**	Page S5
HMBC spectrum of **7**	Page S6
MS spectra of **7**	Page S7
^{1}H NMR spectrum of myxocoumarin B (**9**)	Page S8
MS spectra of **9**	Page S9
^{1}H chemical shift analysis of **9**	Page S10
References	Page S13

^{1}H NMR spectrum (CD_3OD) of myxocoumarin A (**7**)

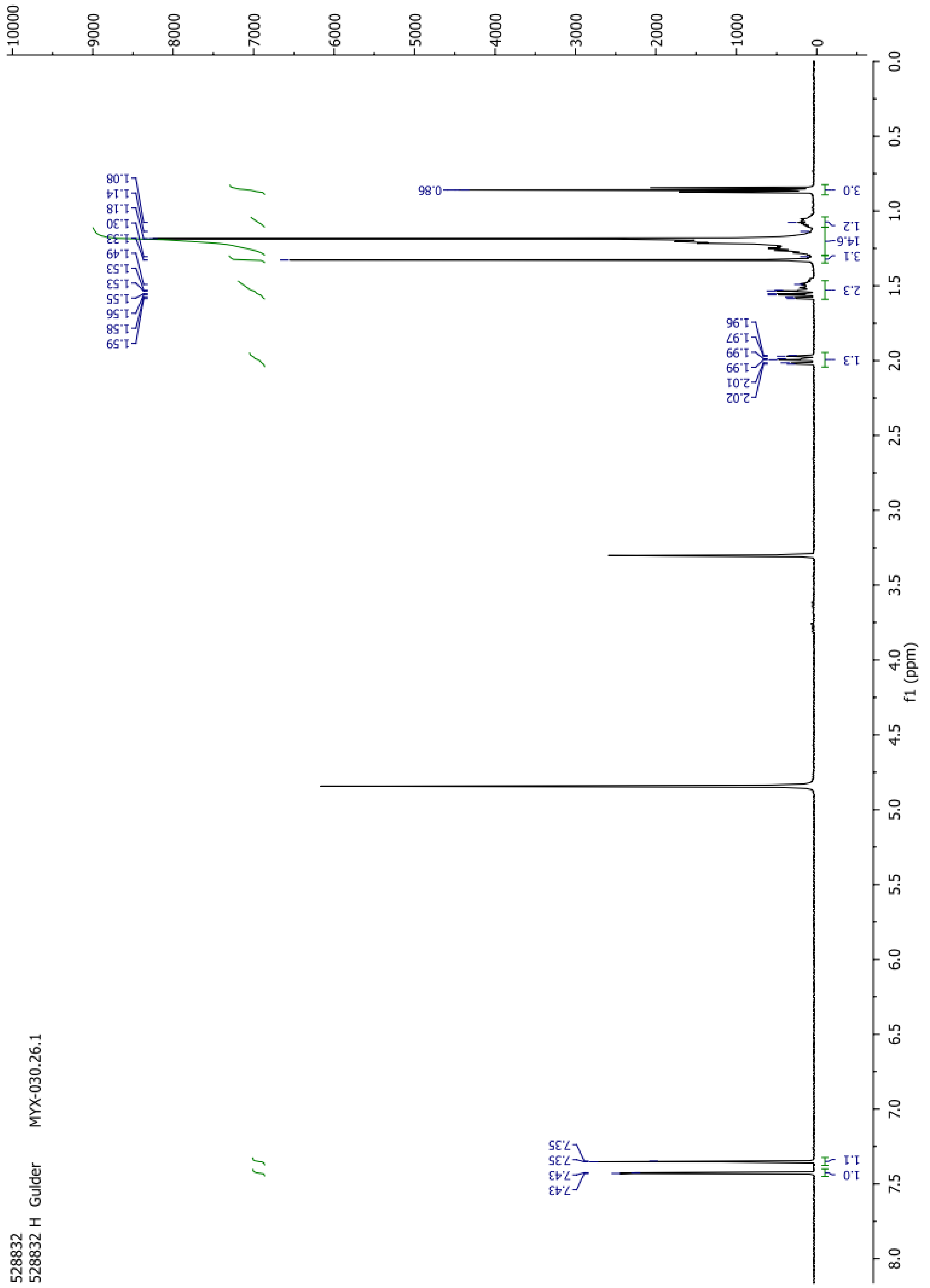

^{13}C NMR spectrum ($CDCl_3$/CD_3OD) of **7**

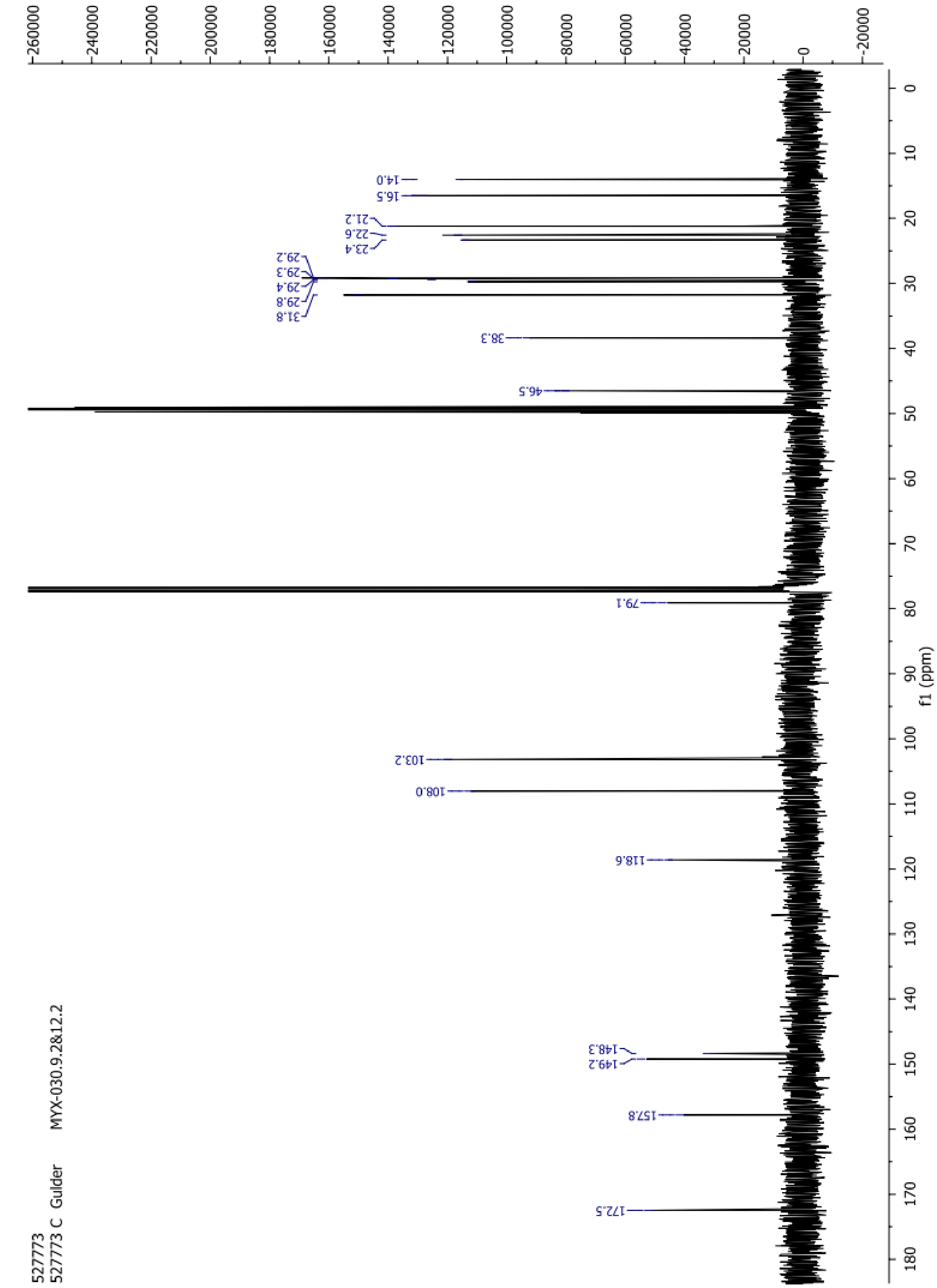

HSQC spectrum (CD_3OD) of **7**

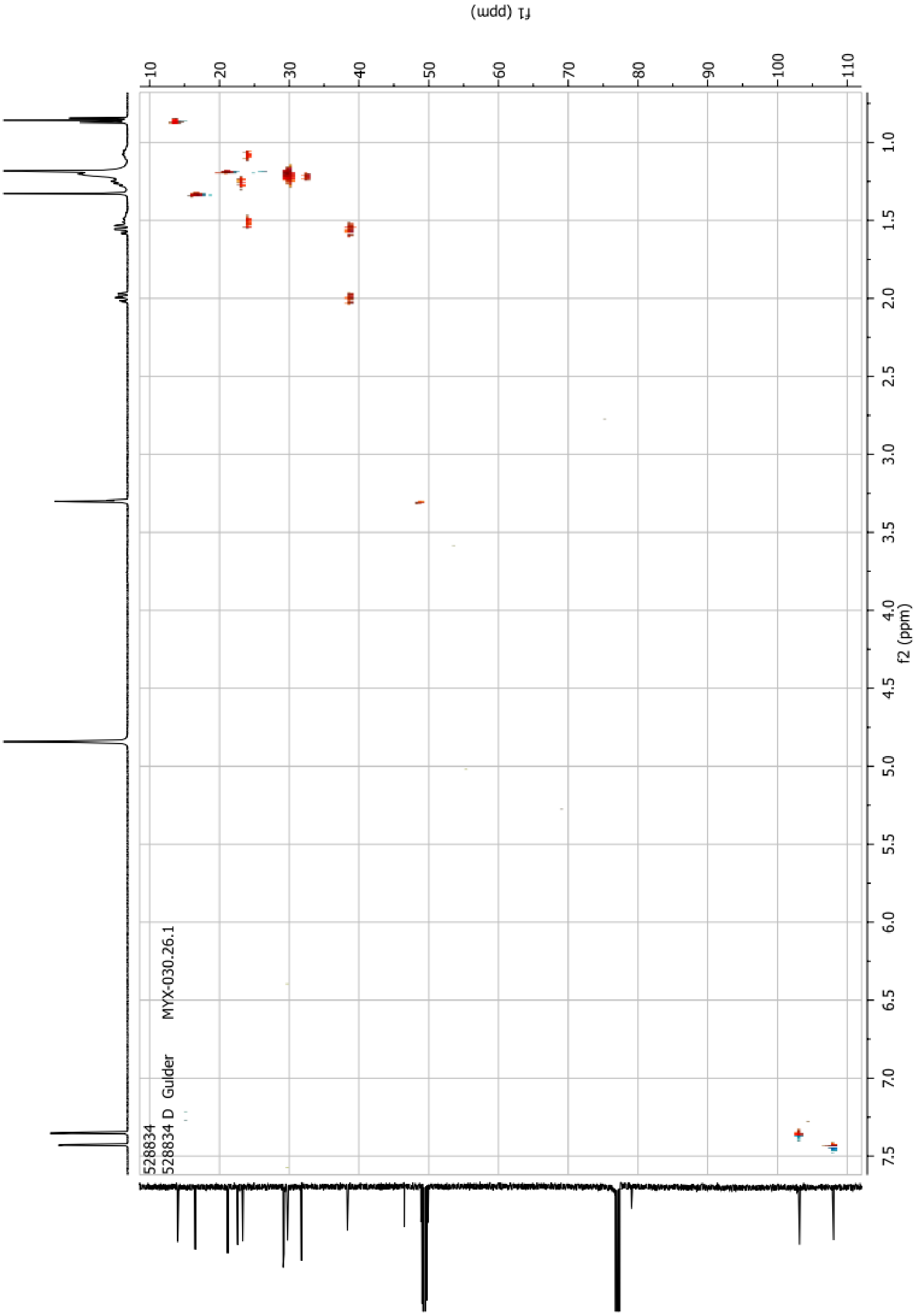

HMBC spectrum (CD_3OD) of **7**

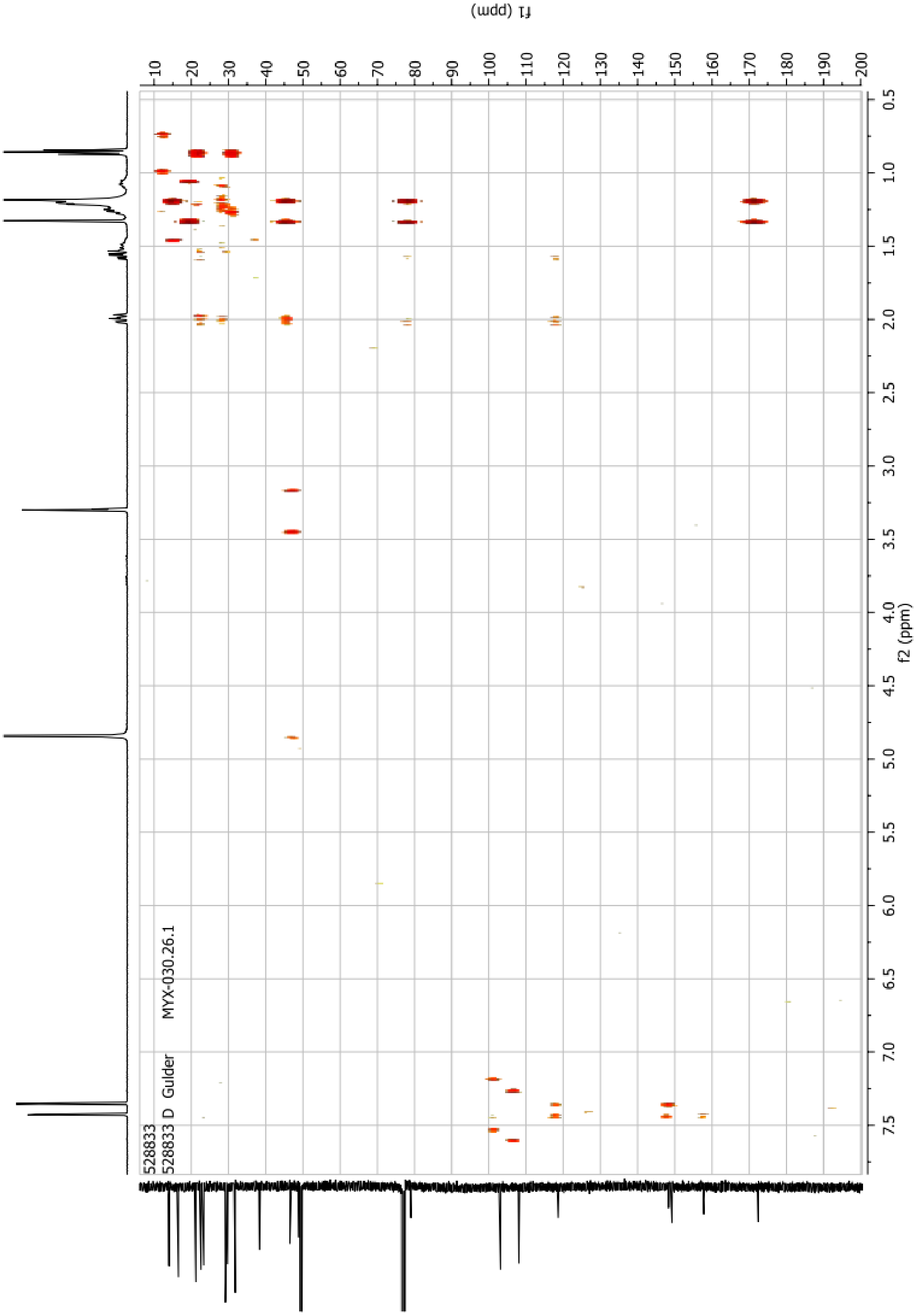

MS spectra of **7**

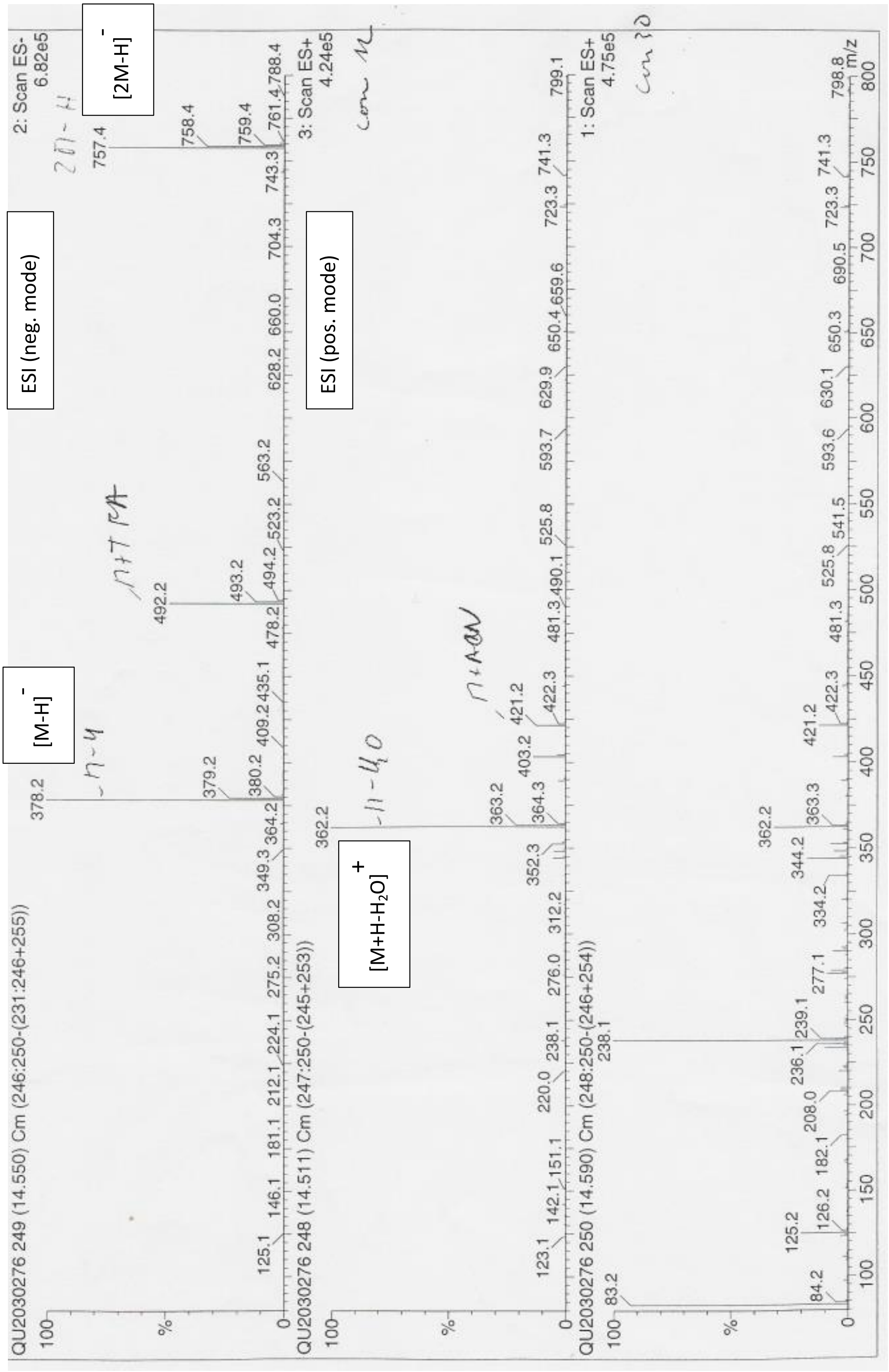

^{1}H NMR spectrum (CD_3OD) of myxocoumarin B (**9**)

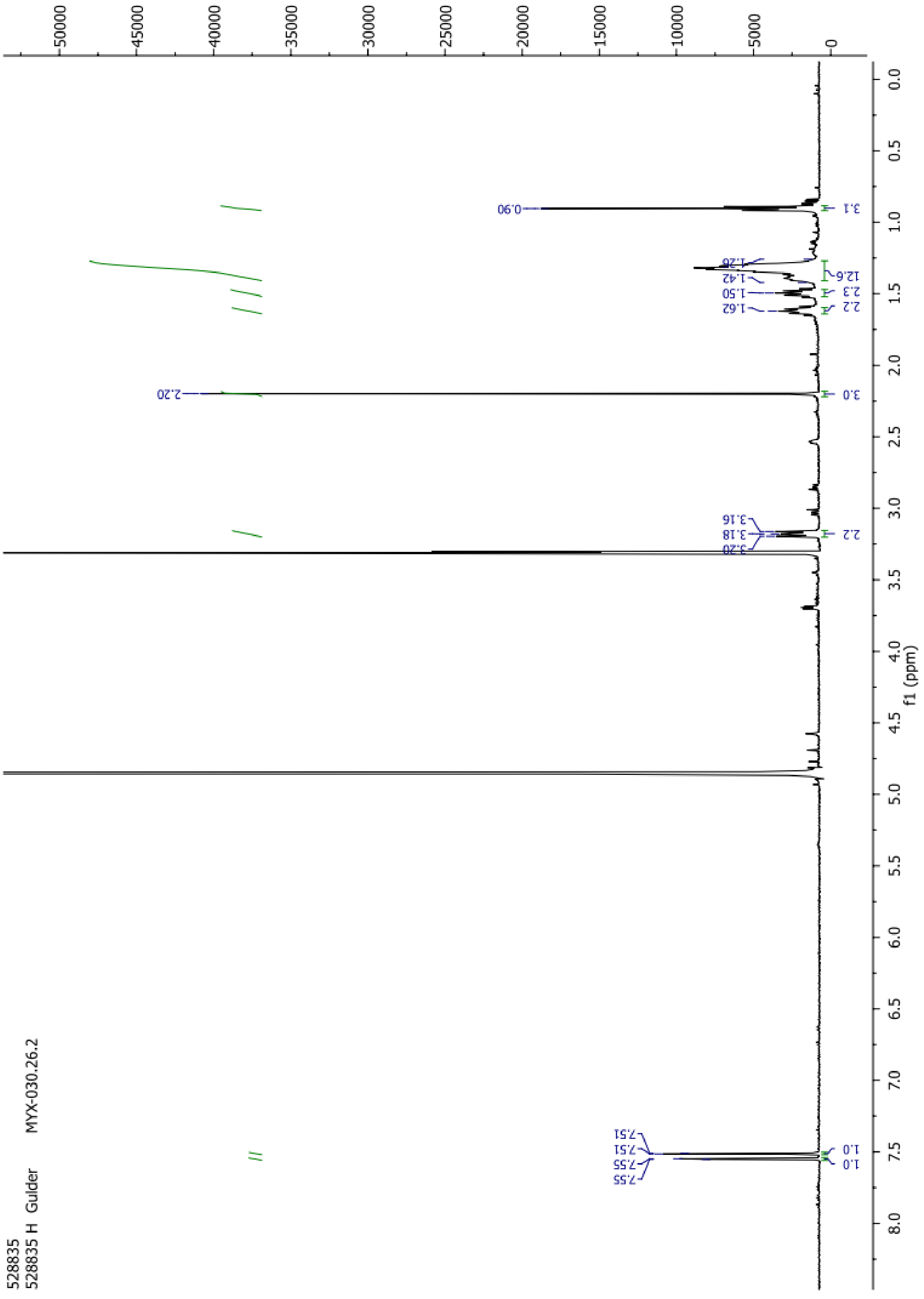

MS spectra of **9**

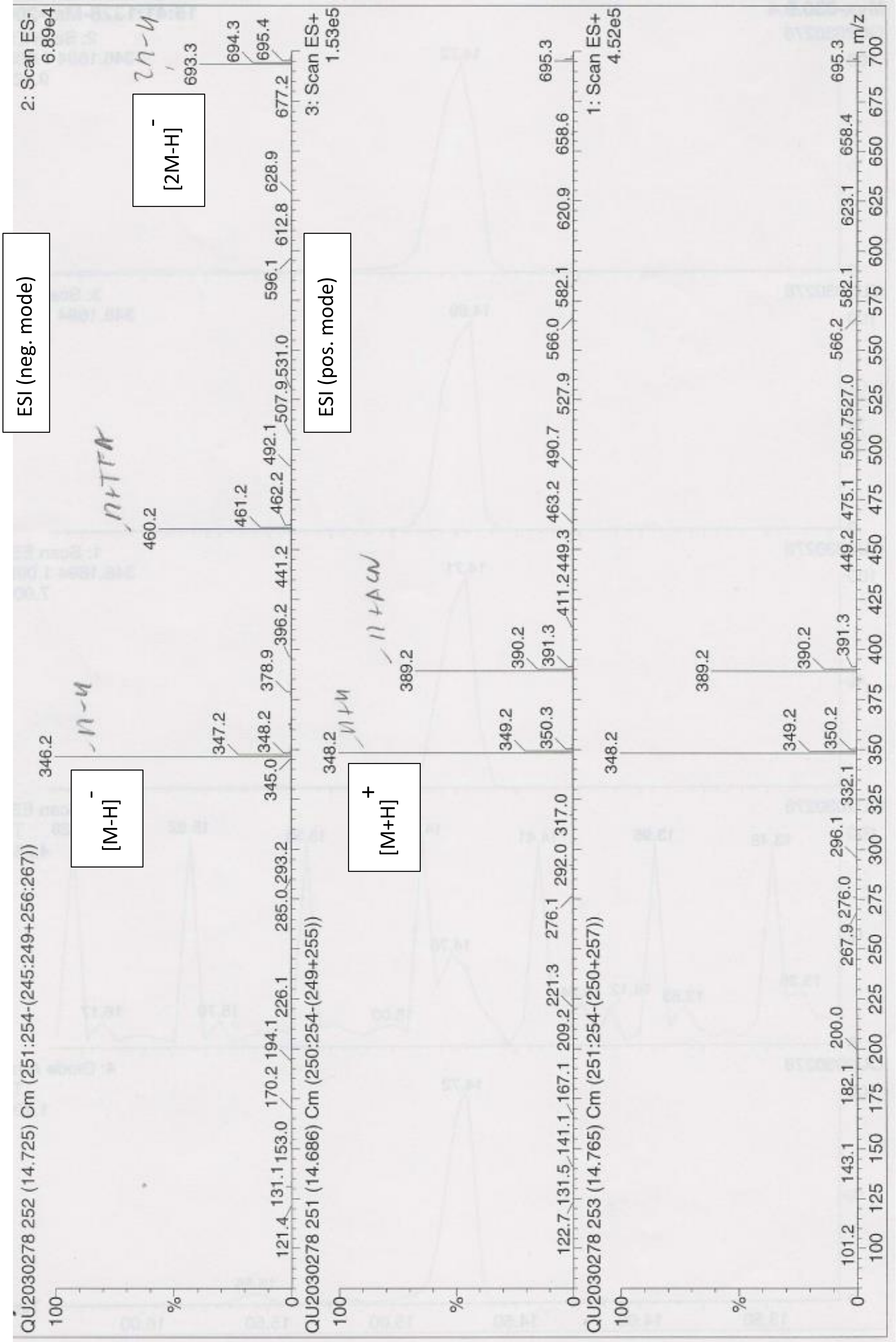

^{1}H chemical shift analysis of myxocoumarin B (9)

Since the available amount of **9** did not suffice to obtain high quality ^{13}C NMR data, the proposed structure of **9** was further corroborated with ^{1}H NMR data from the literature as follows:

In a first step, the identity and positions of the two substituents at the heterocyclic ring were ascertained. Since no benzylic coupling between the aromatic protons and the two alkyl substituents was observed, both alkyl groups had to be located at the heterocycle. Literature data [1,2] shows that a methyl group in 3 or 4 position of a coumarin ring appears at 2.2 or 2.4 ppm, respectively, in $CDCl_3$. Furthermore, a hydroxy group in peri position (at C(5)) to a 4-methyl causes a downfield shift of ca 0.3 ppm of this methyl group, as can be seen by a comparison of such methyl groups in 1-methylnaphthalene (2.65 ppm) [3] and in 8-methyl-1-naphthol (3.00 ppm) [4]. A similar downfield shift is also observed in 4-methyl-5-hydroxycoumarin derivatives [5]. Finally, the chemical shift difference of the benzylic protons in 1-methyl- and 1-ethylnaphthalene [3] (0.43 ppm) as well as 2-methyl- and 2-ethylnaphthalene [3] (0.30 ppm) were used to calculate the influence of the “alkyl chain extension” [6] on the chemicals shifts of the protons at the C atoms directly attached to position 3 or 4 of the coumarin system. Using the three increments extracted above from measured reference data, it is possible to predict the chemicals shifts of the CH_2 groups at position 3 or 4 of the coumarin system starting from the chemical shifts in 3,4-dimethylcoumarin [1]. The result for the two possible isomers is presented in Figure S1. Compound **9** shows the CH_2 group attached to the heterocycle at 3.18 ppm and a methyl singlet at 2.20 ppm (Table S1).

Table S1: ^{1}H NMR spectroscopic data of myxocoumarin B (**9**) at 500 MHz in CD_3OD

position	δ_H (*J* in Hz)^b
6	7.55*, d (2)
8	7.51*, d (2)
9	3.18 m
10	1.62, m
11	1.49, m
12–16	1.26–1.42, m
17	0.90, t (7)
18	2.20, s

*Signal assignment interchangeable

As can be seen in Figure S1, these data are only in agreement with the C_9 alkyl chain in 4-position and the methyl group in 3-position.

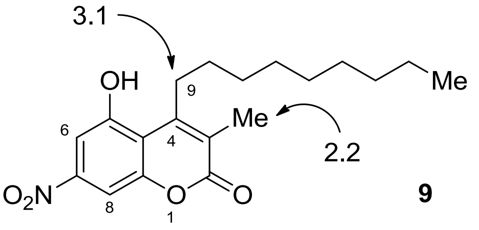

9

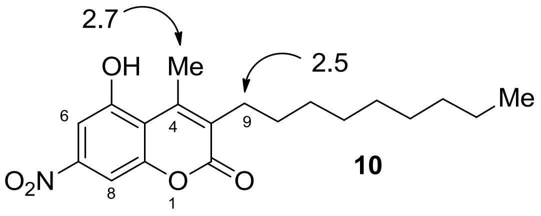

10

Figure S1: Calculated ^{1}H NMR chemical shifts [ppm] for the two conceivable myxocoumarin B isomers **9** and **10**.

In a second step, the chemical shifts of the aromatic protons were compared with the ^{1}H NMR data of the related 5-nitroresorcinol [7]: 7.12 (d, *J* = 2.1 Hz, 2H, H-4, H-6), 6.62 (t, *J* = 2.1 Hz, 1H, H-2). The two aromatic protons in **9** absorb at ca. 0.4 ppm lower than H-4 or H-6 in 5-nitroresorcinol, since the upfield shift caused by the acylated O atom is much smaller than that of a hydroxy group (0.1 ppm [2] as compared to ca. 0.6 ppm [8]). The small chemical shift difference between the two aromatic protons (0.05 ppm) shows that the nitro group occupies the 7-position between these two protons, since the difference of the substituent effects of a nitro

group on the chemical shifts of protons in ortho and para position to it is 0.4 ppm, whereas this difference for the hydroxy substituent is only 0.05 ppm [8].

Finally, the chemical shift of the CH_2 group attached to the 4-position (3.18 ppm) proves that the 5-position is substituted (as discussed above). This concludes the assignment of the structure of **9** by ^{1}H NMR. The resulting substitution pattern given in Figure 3 is thus in full agreement to the structure expected from a joint biosynthetic pathway leading to myxocoumarins A (**7**) and B (**9**) as discussed in the main text of the manuscript.

References

1. D. C. Dittmer, Qun Li, D. V. Avilov, *J. Org. Chem*. **2005**, *70*, 4682-4686.
2. J. B. Rowbotham, T. Schaefer, *Can. J. Chem*. **1973**, *51*, 953-960.
3. C. J. Pouchert, J. Benke, The Aldrich Library of ^{13}C and ^{1}H FT NMR Spectra, Edition I, **1993**.1-methylnaphthalene Vol. 2, 41A, 2-methylnaphthalene Vol. 2, 41B, 1-ethylnaphthalene Vol. 2, 41C, 2- ethylnaphthalene Vol. 2, 42A. The effect of increasing steric crowding on the chemical shift of the methyl group is small (< 0.1 pp) as shown by a comparison of the chemical shifts in toluene (Vol. 2, 1B), o-xylene (Vol. 2, 9A) and 2-ethyltoluene (Vol.2, 9B).
4. F. Gavina, S. V. Luis, P. Ferrer, A. M. Costero, P. Gil, *Tetrahedron* **1986**, *42*, 5641-5648.
5. D. O. Bennardi, D. M. Ruiz, G. P. Romanelli, G. T. Baronetti, H. J. Thomas, J. C. Autino, *Lett. Org. Chem.,* **2008,** *5,* 607-615. B. M. Reddy, B. Thirupathi, M. K. Patil, *Open Catal. J.*, **2009,** *2,* 33-39. N. G. Khaligh, *Catal. Sci. Technol*., **2012**, *2*,1633–1636.
6. The influence of the further CH_2 groups on the chemical shift of the benzylic protons is small, cf. ethylbenzene (2.64 ppm) with 1-phenylnonane (2.60 ppm) in ref. 3, Vol. 2, 1C and 5C.
7. G. Höfle, H. Irschik*, J. Nat. Prod.* **2008**, *71*, 1946-1948.
8. L. Ernst, V. Wray, V.A. Chertkov, N. M. Sergeyev, *J. Magn. Reson*. **1977**, *25*, 123-139.
